# Complete Spontaneous Bone Regeneration following Surgical Enucleation of a Mandibular Cemento-Ossifying Fibroma

**DOI:** 10.1155/2022/7902602

**Published:** 2022-08-05

**Authors:** Saeed Shirafkan, Mehdi Gholamian, Ali Rohani, Sadaf Sadat Mahmoudinezhad, Mahshid Razavi, Kooshan Moradi

**Affiliations:** ^1^Department of Oral and Maxillofacial Surgery, School of Dentistry, Ahvaz Jundishapur University of Medical Sciences, Ahvaz, Iran; ^2^Department of Oral and Maxillofacial Radiology, School of Dentistry, Ahvaz Jundishapur University of Medical Sciences, Ahvaz, Iran

## Abstract

**Background:**

Cemento-ossifying fibroma (COF) is a type of benign fibro-osseous tumor that mainly occurs in the maxillofacial region. Bone reconstruction after the surgery is often performed with bone transplantation. However, the present case report describes the accurate diagnosis and successful surgical resection of a COF with periosteum preservation, after which the defect was completely and spontaneously filled with the newly formed bone through a natural process. *Case Presentation*. A 32-year-old Iranian female patient presented with a history of gradual development of painful swelling, spontaneous pain, and lower lip and chin hypoesthesia in the lower third of the left side of her face. The dome-shaped swelling was tender. The patient was suffering from renal infection and urethral prolapse and was taking folic acid. She also mentioned a positive family history of similar swellings in her mother and uncle. Intraoral examination indicated a lesion in buccal and lingual vestibules extending from the first premolar to the third molar teeth. It had a firm consistency, and the covering mucosa was normal in terms of color and texture. The aspiration test was negative. The lesion had caused severe mobility of the second premolar and first and second molar teeth. Panoramic radiography revealed an extensive well-defined unilocular radiolucency. Significant knife-edge resorption of the first and second molar roots at the involved site and thinning of the alveolar crest and inferior border of the mandible were also clear. Cone-beam computed tomography showed severe expansion in the buccal and moderate expansion in the lingual aspect, causing thinning of both the buccal and lingual cortical plates. Histopathological analysis revealed neoplastic tissue mixed with fibrous connective tissue and several round and oval-shaped calcification foci. Immunohistochemical analysis confirmed the final diagnosis (COF) with the presence of SMA-8. The lesion was removed by enucleation and curettage, while the periosteum was carefully preserved. Fixation with screw and plate was also performed.

**Conclusions:**

Correct diagnosis of COF and precise implementation of the periosteal osteogenesis technique, in this case, resulted in entirely and spontaneously bone regeneration, which was a rare and favorable outcome with minimum cost and complications for the patient.

## 1. Background

Cemento-ossifying fibroma (COF) is a benign fibro-osseous tumor that mainly involves the maxillofacial region [1]. This neoplastic lesion has a capsule and is almost well defined. It contains a mixture of fibrous tissue and different amounts of calcified tissues resembling bone, cementum, or both [2, 3]. COF was first categorized as a fibro-osseous neoplasm by the World Health Organization [4]. It often occurs in the second to fourth decades of life and is more common in females [3]. It does not involve the long bones; instead, it commonly occurs in the tooth-bearing areas of the jaws [5]. The mandible, particularly at the molar–premolar region, is the most common location for the occurrence of COF [6]. It originates from the mesenchymal cells, which are found in the periodontal ligament and capable of forming fibrous tissue, cementum, bone, or a combination of all [2, 7]. Most COF lesions have a slow growth pattern and often remain undetected by the patient until swelling of the face becomes prominent. However, in rare cases, COF may grow rapidly and cause clinical symptoms [2]. The incorrect surgical technique could lead to relapse. Thus, correct diagnosis and treatment plan can lead to optimal predictable results in the management of COF [8]. Herein, we report the diagnostic workup (which included radiographic, histopathological, and immunohistochemical assessments), and successful surgical resection of a COF. Following surgery, the bone defect was completely filled with the newly formed bone through a spontaneous natural process.

## 2. Case Presentation

This retrospective study was approved by the ethics committee. The study was performed in accordance with the ethical standards as laid down in the 1964 Declaration of Helsinki. The patient gave her informed consent prior to use of her medical reports and images in the present case report study. The present manuscript is written following CARE guidelines.

A 32-year-old female patient presented to a dental clinician complaining of gradual development of painful swelling in the lower third of the left side of her face. After the initial examination, the dental clinician referred her to the Oral and Maxillofacial Surgery Department.

### 2.1. Extraoral Examination

Extraoral examination revealed a dome-shaped swelling in the lower third of the left side of the face. The skin over the involved site had normal color and texture. The lesion was tender on palpation, and the patient reported spontaneous pain and hypoesthesia of the lower lip and chin toward the left side. The patient also declared suffering from renal infection and urethral prolapse and was taking folic acid. She also mentioned a positive family history for such lesions (in her mother and uncle).

### 2.2. Intraoral Examination

Intraoral examination indicated a lesion in the mandibular left side buccal and lingual vestibules extending from the first premolar to the third molar teeth. It had a firm consistency, and the covering mucosa was normal in terms of color and texture. The aspiration test was negative. The lesion had caused severe mobility of the second premolar and the first and second molar teeth.

### 2.3. Imaging Findings

**Figure 1:**
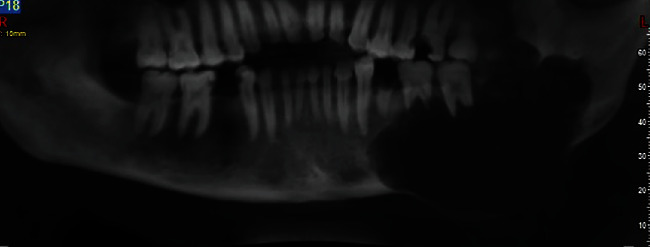
Panoramic view upon presentation.

Panoramic radiography revealed an extensive well-defined unilocular radiolucency in the body of the mandible at the left side, extending from the apex of the first premolar of the same side to the upper third of the mandibular ramus horizontally, and from the alveolar crest to the inferior border of the mandible vertically. It had caused severe expansion toward the mandible's inferior border and the alveolar crest (Figure 1). Significant knife-edge resorption of the first and second molar roots at the involved site and thinning of the mandible's alveolar crest and inferior border were also evident.

**Figure 2:**
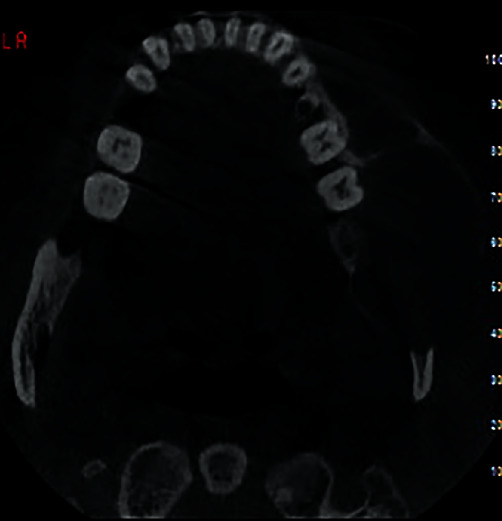
Axial CBCT section upon presentation.

**Figure 3:**
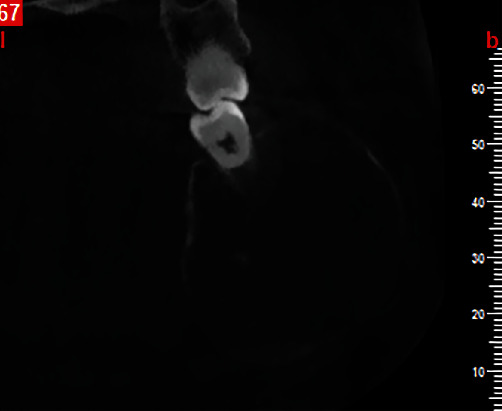
Cross-sectional CBCT image upon presentation.

**Figure 4:**
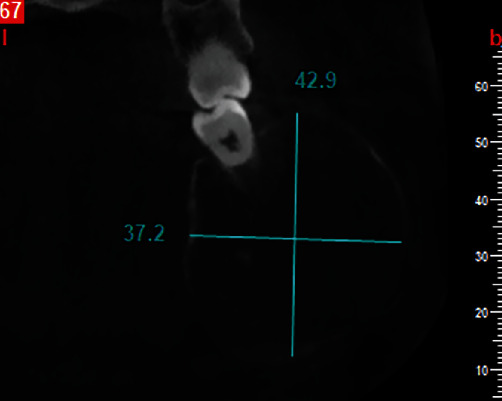
Labiolingual and superoinferior size of the lesion on cross-sectional CBCT section. (labiolingual size = 37.2 mm, superoinferior size = 42.9 mm).

**Figure 5:**
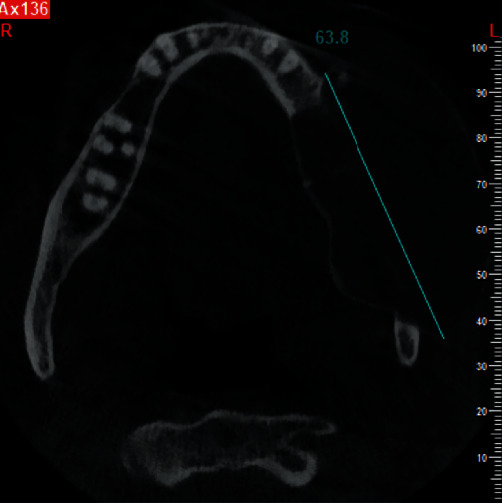
Anteroposterior size of the lesion in axial CBCT section.

Cone-beam computed tomography was then performed. The axial and cross-sectional views indicated severe expansion in the buccal and moderate expansion in the lingual aspect, causing thinning of both the buccal and lingual cortical plates (Figures 2 and 3). Labiolingual and superoinferior lesion sizes on the cross-sectional CBCT section were 37.2 mm and 42.9 mm, respectively (Figure 4). Also, the anteroposterior size of the lesion in the axial CBCT section was 63.8 mm (Figure 5). Some calcification foci were also noted in the radiolucent lesion. According to the radiographic findings, the list of differential diagnoses included ossifying fibroma, central giant cell granuloma, and aneurysmal bone cyst.

### 2.4. Histopathological Findings

Histopathological analysis of the enucleated lesion was performed by an oral and maxillofacial pathologist. The pathology report disclosed a lesion with cream color and firm consistency measuring 3 × 4 × 5 cm, which was associated with 3 teeth. Microscopic assessments revealed neoplastic tissue mixed with fibrous connective tissue and several round and oval-shaped calcification foci. The primary diagnosis by the pathologist was spindle cell neoplasm, which is a malignancy in the list of differential diagnoses with many other benign and malignant lesions. Since the radiographic findings indicated that the inferior border of the mandible had not been invaded or perforated, and was only displaced due to severe expansion of the lesion, the pathological diagnosis of spindle cell neoplasm was questioned by the surgeon, because malignancies often invade the border and cause destruction of cortical bone. Thus, immunohistochemical analysis was requested, which was positive for smooth muscle actin (SMA-8). The presence of this marker led to a definite diagnosis of COF.

### 2.5. Surgical Procedure

**Figure 6:**
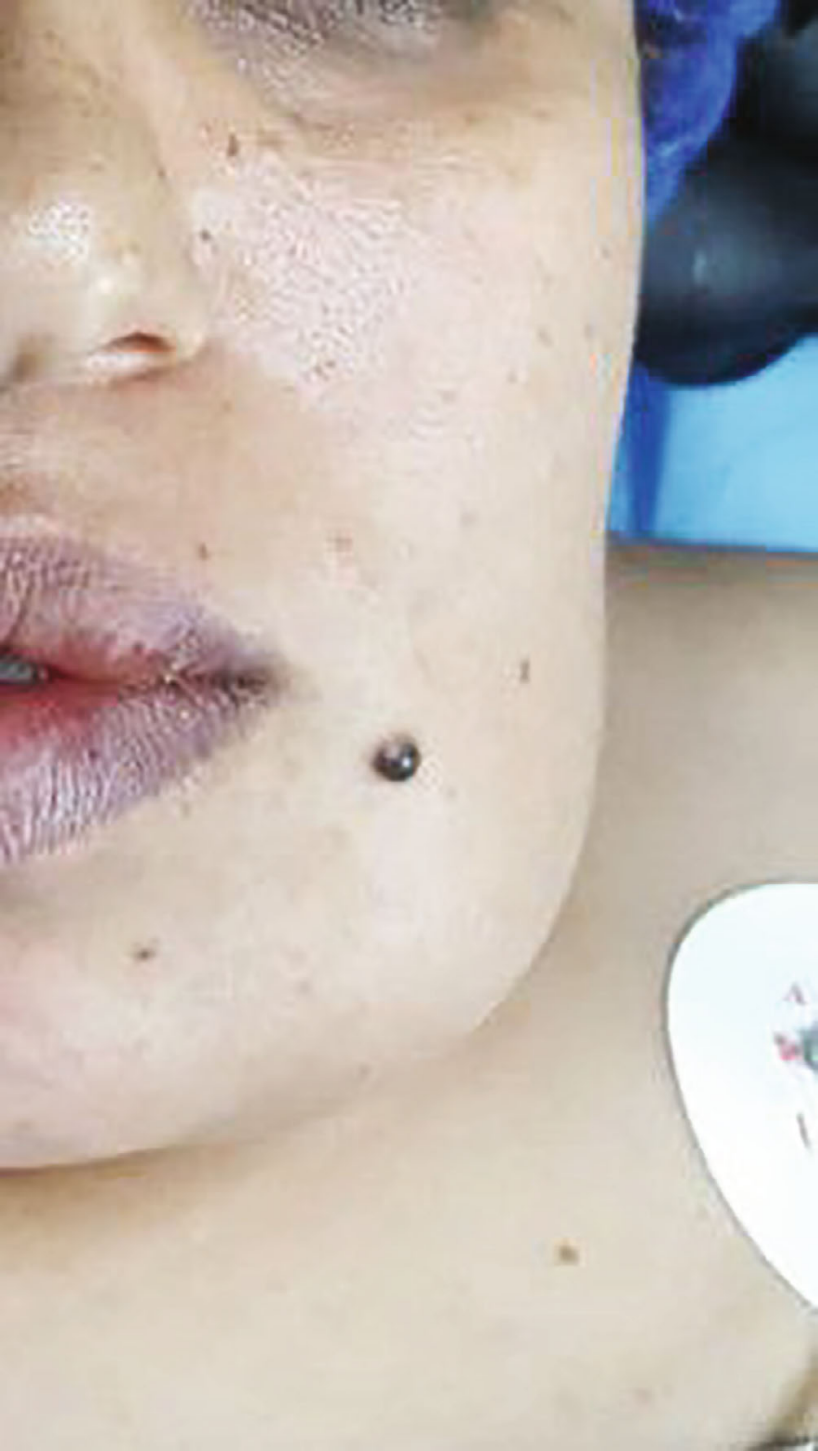
Extraoral preoperative view.

**Figure 7:**
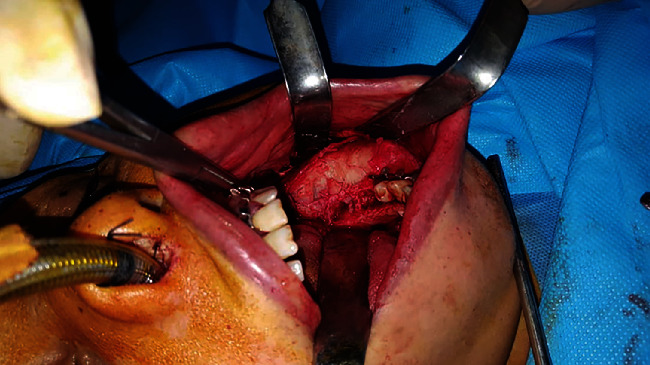
Intraoral intraoperative view.

**Figure 8:**
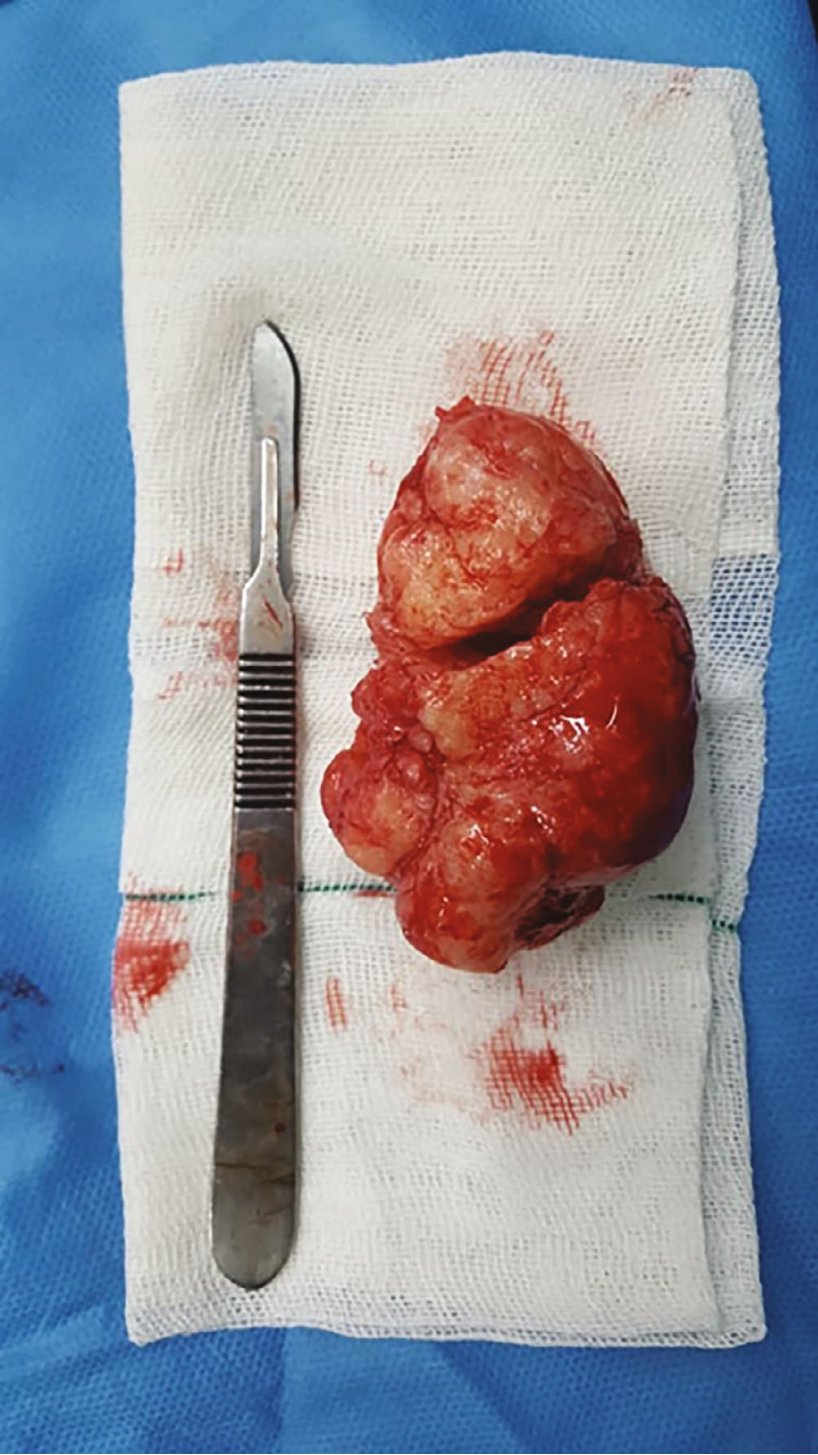
Enucleated lesion.

**Figure 9:**
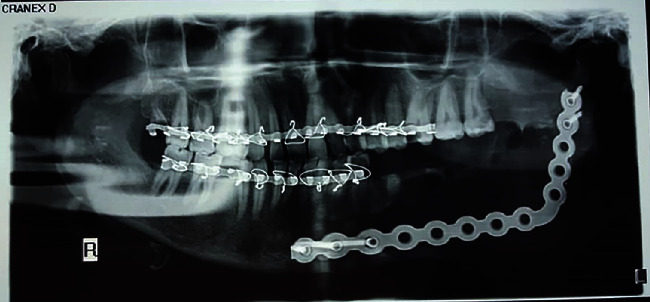
Panoramic view at 1 week postoperatively.

**Figure 10:**
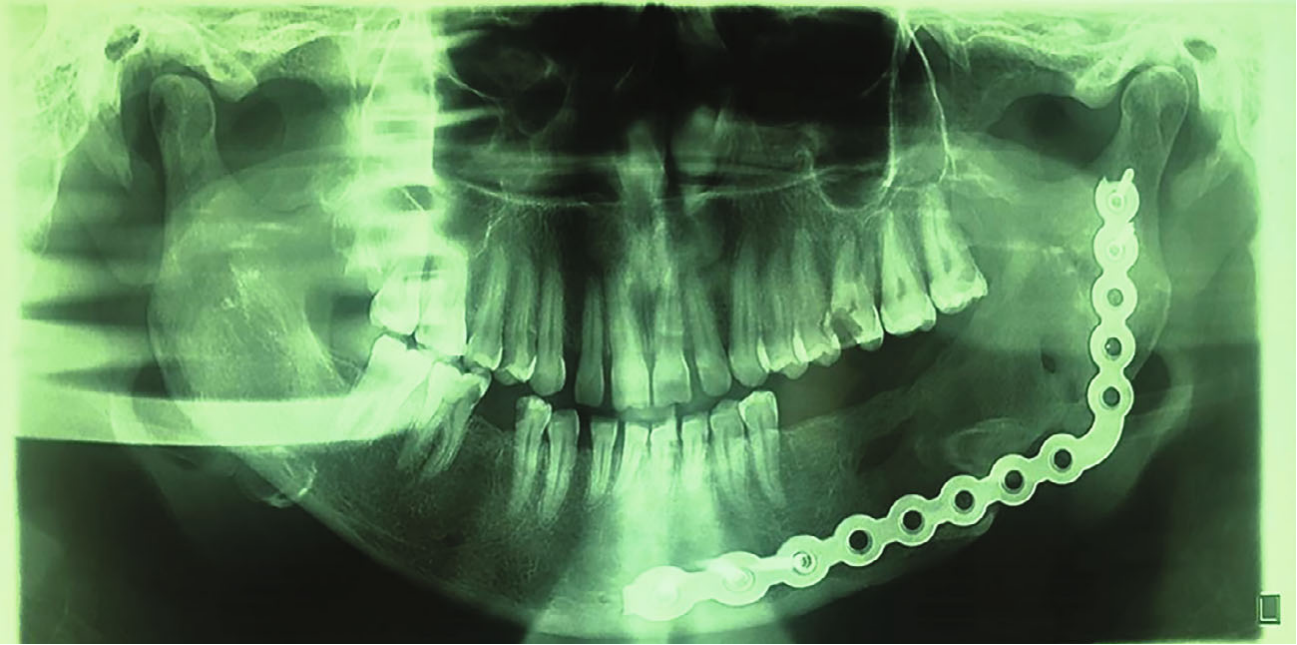
Panoramic view at 1 year postoperatively, indicating spontaneous bone fill of the defect.

**Figure 11:**
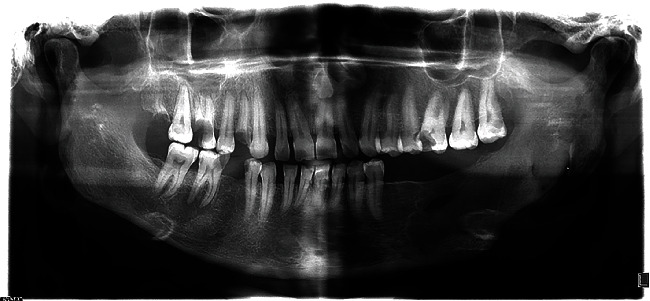
Panoramic view at 3 years postoperatively, indicating spontaneous bone fill of the defect.

**Figure 12:**
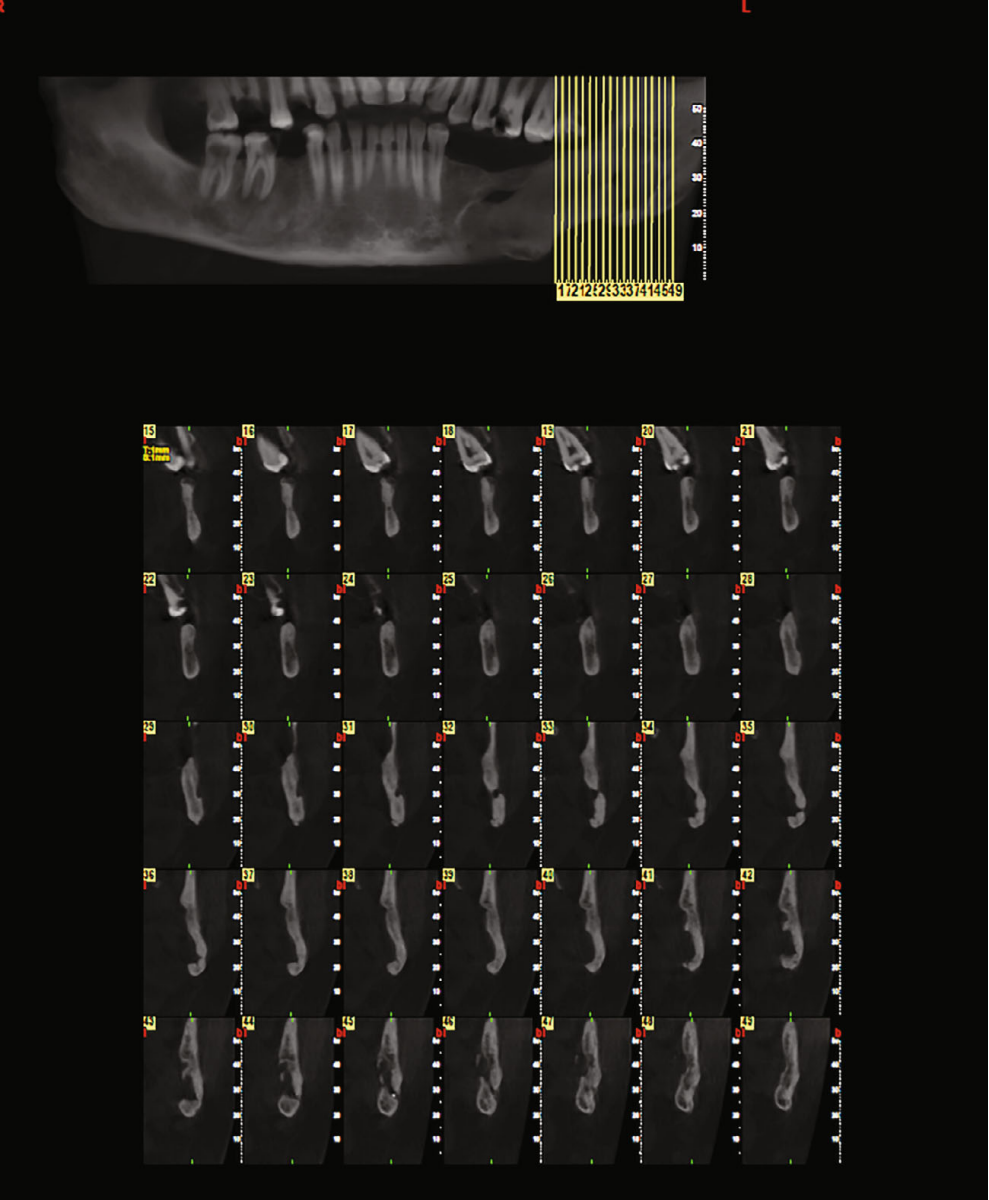
Cross-sectional CBCT images at 3 years postoperatively, indicating spontaneous bone fill of the defect (after ensuring complete spontaneous bone fill of the defect, the plate was removed in another surgical procedure).

**Figure 13:**
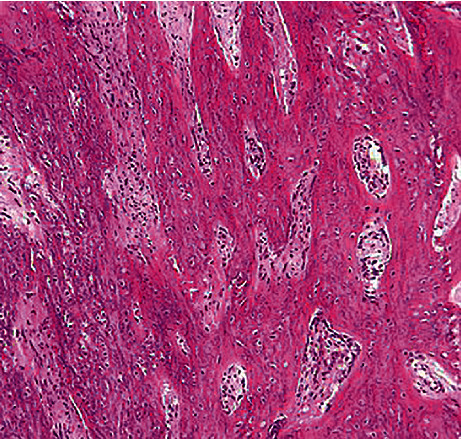
Well-shaped trabecular bone and bone marrow space filled with dense fibrous connective tissue.

Surgical enucleation was performed 9 months after the development of the lesion. Precise diagnostic workup revealed the benign nature of the lesion. Thus, to adopt the most conservative approach for minimal removal of intact bone and also to benefit from the inherent regenerative potential of the periosteum, it was decided to perform enucleation and curettage. Accordingly, the submandibular or Risdon approach was adopted to access the lesion after disinfecting with chlorhexidine and povidone-iodine [9]. First, a mark was made on the skin 3 cm below the inferior border of the mandible to preserve the marginal mandibular nerve. The local anesthetic agent was then administered. After gentle and careful elevation of the periosteum (to preserve and protect it), the tumor was resected subperiosteally with 1 cm of safe margin both anteriorly and posteriorly (considering its benign nature and also for maximum preservation of healthy bone tissue) (Figures 6–8). A reconstruction plate was then adapted and installed to reinstate the occlusion. After enucleation of the tumor and curettage, the area was copiously irrigated. The incision was then sutured after approximation of the dissected layers. Arch bar and elastics were then placed. The follow-up sessions were scheduled at 1 week, 3 months, 6 months, 1 year, and 3 years postoperatively (Figures 9–12). After 1 year from surgery, which was the time for bone grafting, a panoramic radiograph was requested, which revealed complete bone fill of the defect. Also, 3 years after the surgery, cross-sectional CBCT images revealed complete bone regeneration and no recurrence of the COF (Figure 12). The histopathological evaluation of the regenerated bone was also assessed. An incisional biopsy was obtained during plate removal, and the sections were stained with hematoxylin and eosin.  Figure 13 shows a well-shaped trabecular bone and bone marrow space filled with dense fibrous connective tissue, which indicates spongy bone histology.

The skin of the lip corner, chin, and face of the involved side was also carefully examined, which revealed paresthesia. After ensuring complete spontaneous bone fill of the defect, the plate was removed in another surgical procedure.

## 3. Discussion

Oral and maxillofacial surgeons are searching for ways to functionally and esthetically reconstruct the mandibular bone defects caused by the resection of lesions. However, the available approaches for the reconstruction of mandibular bone defects are complex and challenging. Recently, oral and maxillofacial surgeons have been more interested in benefiting the spontaneous bone regeneration capacity of the bone, which is likely to happen with the preservation of the periosteum after mandibulectomy. To reinstate the normal morphology and function of the lost or injured tissue, it would be ideal to benefit from the inherent healing and regenerative potentials of the human body in reconstructive procedures. Therefore, this topic has gained increasing popularity in regenerative medicine. This approach has numerous advantages for reconstruction of mandibular bone defects such as reduction of costs and postoperative complications. However, such advantages should be weighed against the possibility of shortcomings such as unpredictability of the amount of formed bone in some cases [10–12].

To induce spontaneous bone regeneration, the periosteum should remain intact to serve as a natural source of viable bone cells and provide the necessary vascular support. Also, the periosteum should be able to serve as a barrier against the migration of soft tissue into the defect to allow osteogenesis [13–17], which was precisely performed in the present case.

Assessment of more than 600 cases over 20 years in a retrospective study revealed that following regional resection of the mandible, the possibility of spontaneous bone regeneration would only be 2%. The present case was among the aforementioned 2% fraction in whom spontaneous bone regeneration resulted in complete bone fill of the defect [13, 18].

The mechanism of action of the periosteal osteogenesis involves angiogenesis and neurogenesis [10, 19, 20]. The reason behind angiogenesis in the process of bone regeneration is obvious. However, with regard to neurogenesis, particularly in the maxillofacial region, it has been discussed that the expression of genes related to neurogenesis has a positive effect on the promotion of osteogenesis in this region. Some studies have reported a controlling mechanism through which the bone regeneration process is regulated by the central nervous system [10, 21].

The younger patient's age, stability of the defective segment following fixation with plate and screw, and the absence of infection are among the factors that affect the process of new bone formation at the defect site. In the present case, although the patient had a positive history of renal infection, spontaneous osteogenesis happened appropriately without additional need for a bone graft [13, 17, 22].

According to the textbook of *Oral and Maxillofacial Pathology* by Neville and colleagues, mutations in the HRPT2 tumor suppressor gene were recently detected in two patients with different disorders such as renal cysts and COF of the jawbone. Considering the positive history of renal infection in our patient, mutation of the abovementioned gene may be postulated [23].

Evidence shows that the bone marrow stem cells, which are currently used for cell therapy approaches in orthopedics for regeneration of bone and cartilage, have lower regenerative potential than the periosteum-derived cells for regeneration of bone and cartilage [24, 25].

A study conducted in 2020 on bone regeneration by the periosteum progenitors indicated that a type of mesenchymal stem cells known as Prx1^+^ cells that are present in the periosteum actively participate in the process of bone regeneration of defects. The number of these cells significantly decreases with aging, which results in impairment of the regenerative potential of bone over time. Also, they confirmed that Prx1^+^ mesenchymal stem cells can enhance early healing of bone defects [24]. Moreover, some other studies have documented that periosteum-derived Nestin^+^ and LepR^+^ cells located in the outer layer of the periosteum are capable of regeneration and chemotaxis of bone progenitor cells [26, 27]. Unlike the Prx1^+^ cells that are exclusively found in the skeletal system, the Nestin^+^ and LepR^+^ cells are present in other tissues as well [24, 28, 29]. Furthermore, it has been discussed that the bone morphogenetic protein 2 (BMP2) also serves as an initial regulator of periosteal bone regeneration. The BMP2 is famous for its role in skeletal growth and development and is believed to have an inherent potential to induce bone regeneration. The target cells of BMP2 are located in the periosteum. Also, it has been claimed that maximum therapeutic efficacy can be achieved by benefitting from the recombinant form of BMP2 to increase the density of the newly formed bone in the process of periosteal bone regeneration [30–32].

Recently, a protein known as periostin was discovered, which is synthesized in response to mechanical stress, injuries, and pathological conditions [25, 33–35]. In different cancer types, particularly the common high-risk cancers such as the lung cancer, elevated levels of periostin are associated with poor prognosis and metastasis [36]. In addition, a considerable increase in the level of periostin in periosteal cells has been reported in skeletal injuries, highlighting its role in optimal regeneration of the injured bone [25].

Review of the recently published articles indicates the occurrence of periosteal osteogenesis not only in the maxillofacial region but also in other parts of the human skeleton. In 2020, Rajak et al. [37] reported a case of an aneurysmal bone cyst in the clavicle. They reported bone regeneration induced by the intact periosteum after marginal resection of the lesion on the first follow-up.

In 2020, Colangeli et al. [38] reported a 12-year-old boy with Ewing's sarcoma in the ulnar diaphysis. The patient underwent an extensive resection of the ulnar diaphysis, and then an ipsilateral fibula graft was harvested as an autologous non-vascularized graft. The periosteum was carefully preserved in the process of bone harvesting. Radiographic examinations of the donor site performed in the first six months after surgery indicated complete bone regrowth and regeneration at the graft harvesting site. Some studies have discussed that chemotherapy may damage the periosteum and impair its bone regenerative potential. However, in their case, despite the conduction of chemotherapy, the periosteum preserved its regenerative capacity. According to the literature, younger age and the presence of a thicker periosteum increase the possibility of faster and more extensive bone regeneration [38–41].

COF may manifest different radiographic patterns depending on its degree of mineralization. Initially, it manifests as a radiolucent lesion. The calcified foci gradually increase as the lesion matures until a completely radiopaque appearance is created [6, 42, 43]. The main characteristic feature of COF is its eccentric growth pattern, which results in its expansion in all directions and eventual formation of a prominent spherical mass, which is easily differentiable from the adjacent tissues [3, 43]. Titinchi and Morkel [34] showed that in approximately 50% of the cases, COF is radiopaque. It manifests as a combined radiolucent–radiopaque lesion in 35%, and purely radiolucent lesion in only 15% of the cases; our case was categorized in the latter group.

According to the existing literature on this topic, clinically, COF is often painless and appears as a slow-growing mass in the jaw with tooth displacement as its first symptom. Moreover, the teeth at the site remain vital, and root resorption rarely occurs [44, 45]. However, in the present case, the lesion had caused root resorption without causing tooth displacement. Root resorption was reported in 12% of the cases in the study by Titinchi and Morkel and the majority of COFs causing root resorption were multilocular, and occurred in young patients. However, the lesion in our patient was unilocular and caused root resorption of the teeth at the site [46].

The well-defined margin of COF aids in its differentiation from invasive sarcomas and carcinomas. Accordingly, the attending surgeon of the present case suspected the accuracy of the initial pathological diagnosis regarding the lesion being a spindle cell neoplasm and requested immunohistochemical analysis [47, 48]. COF often expands without perforating the surrounding cortical bone and forms a distinct spherical shape [47].

Since COF can be clearly differentiated from the adjacent bone, it can be resected conservatively. The suggested treatment plans include enucleation for small lesions, curettage for the lesions with no distinct radiolucency around them, and en bloc resection along with bone regeneration for large lesions close to the inferior border of the mandible [46].

Due to resistance to radiation, radiotherapy of COF would be complicated. It has a good prognosis although the risk of relapse in the maxilla is much higher than that in the mandible due to greater complexity of the surgical procedure in the maxilla particularly for large lesions [49]. Complete and precise surgical resection of the lesion at the earliest time possible has been widely advocated [46].

## 4. Conclusions

Finally, it should be noted that precise diagnosis of the lesion in the present case and correct adoption of periosteal bone regeneration approach resulted in complete bone regeneration, which was a rare and favorable outcome with minimum cost and complications for the patient.

## Data Availability

The data used to support the findings of this study are available from the corresponding author upon request. (Contact details: sadafmahmoudinezhad@gmail.com).

## Abbreviations

COF: Cemento-ossifying fibroma
SMA: Smooth muscle actin
